# Factors and Contingencies for the “It Pays to Be Green Hypothesis”. The European Union’s Emissions Trading System (EU ETS) and Financial Crisis as Contexts

**DOI:** 10.3390/ijerph16162988

**Published:** 2019-08-20

**Authors:** Joaquín Cañón-de-Francia, Concepión Garcés-Ayerbe

**Affiliations:** Department of Management, University of Zaragoza, 50005 Zaragoza, Spain

**Keywords:** environmental proactivity, financial performance, contingent theory, European Union’s Emissions Trading System, financial crisis

## Abstract

This study provides empirical evidence related to the “it pays to be green” hypothesis. Based on information from panel data approximately 42 industrial companies during an 8-year period, we determine some of the factors and contingences that affect the fulfilment of that hypothesis. We find that a certain level of proactivity in environmental strategy design is one of the conditions that favors a positive relationship between environmental investment and financial performance. We also provide empirical evidence on how some external conditions affect this positive relationship, such as regulatory pressure from the European Union Emissions Trading System (EU ETS) and the financial crisis.

## 1. Introduction

Regulatory pressure aimed at firms has increased over the past few years, particularly in the United States and European Union. Adaptation costs for firms have been very high, and many of them have attempted to integrate their environmental efforts into their business strategy.

Drawing on a resource-based view, many studies have analyzed environmental strategy as an element that improves competitiveness. From this perspective, several authors such as Hart [1], Hart and Ahuja [2], Russo and Fouts [3], Judge and Douglas [4], Sharma and Vredenburg [5] and Hart and Dowell [6] have provided the theoretical framework for a large number of empirical studies that do not provide conclusive results about the “it pays to be green” assumption. Thus, it might be concluded that empirical evidence is contradictory.

Indeed, although many studies have found that environmental protection investment is linked to positive economic performance [7,8,9,10,11,12,13,14,15], there is also empirical evidence that shows that any further effort to improve environmental performance ineluctably yields lower profits [16,17,18,19,20,21,22].

In an attempt to shed light on this debate, business-organization academics have focused on studying the conditions and contexts in which it pays to be green. Three different lines of research have been presented in the literature to explain the lack of consensus regarding the economic repercussions of environmental management. The first maintains that environmental management can be of different forms, and that the chosen form conditions the likelihood of achieving positive economic outcomes. Several empirical studies by Klassen and Why bark [23], Christmann [24], King and Lenox [25], González-Benito and González-Benito [26], Guenster et al. [10], Aragón-Correa et al. [27], Sambasivan [28] and Ghisetti and Rennings [29] have shown that the nature of environmental strategies, as well as varying the degree of corporate environmental proactivity (defined as the firm’s tendency to go beyond legal requirements and institutionalized environmental practices in the industry), will determine the likelihood of said strategy having a positive impact on corporate economic results.

Another reason for the conflicting empirical results in research on the relationship between environmental and economic performance is that internal company resources and general business environments condition the economic returns of environmental investment. In this regard, the contingency theory has provided a theoretical framework for explaining how external context variables condition the relationship between environmental initiatives and financial performance. This contingency approach has been followed by authors such Aragón-Correa and Sharma [30] and Aguilera-Caracuel and Ortiz [31]. 

According to the third argument, the heterogeneous evidence in the study of the “it pays to be green” hypothesis can be due to the different methodological criteria used in the analysis, such as how variables are measured, the period considered, sample size or estimation methods (King and Lenox [8],Ambec and Lanoie [32], Busch and Hoffmann [33],Trumpp and Guenther [34]).

Considering these three types of argument, this paper analyzes the impact of environmental protection measures on economic performance. It begins by emphasizing the environmental proactivity concept, defined as the tendency to prevent (rather than correct) pollution, with environmental protection practices that go beyond mere obedience to the law and regular industry practices. It then considers the impact of different external contingencies, such as regulatory pressure from the European Union Emissions Trading System (EU ETS) and the financial crisis. Thirdly, the selection and design of the variables and the estimation methodology solve some of the methodological problems identified in the literature.

Some of the main contributions of this study relative to previous literature on the topic are a persuasive and objective measure of a firm’s efforts regarding environmental protection. Unlike most previous studies, we designed environmental proactivity from objective secondary information: firms’ investments in pollution control and prevention technologies. On the other hand, we calculated our financial results through an objective measurement that considers firms’ future streams of earnings, incorporating the expected long-term profits of environmental investments. Consideration of the effects of external contingencies on the analysis also adds to the results of this research.

The application of a panel data model is another differentiating point of this study, as this methodology can explicitly take into account individual heterogeneity by incorporating the impact of specific effects of a company on endogenous variables (associated with knowledge, culture, resources and individual capabilities, etc.).

This study includes six sections. The section that follows this introduction reviews the literature that characterizes environmental proactivity as a mechanism for improving competitiveness. The third analyses the contingent variables that can influence environmental proactivity. The fourth presents the methodology. The fifth section presents the results obtained in the empirical analysis. Finally, the main conclusions are summarized in the sixth section.

## 2. Economic Consequences of Environmental Proactivity: The Resource-Based View Perspective 

Porter [35], Porter and Van der Linde [36,37], Esty and Porter [38] and others have argued that properly designed environmental regulation should not been seen only as a burden for companies, but as something capable of triggering innovation in the search for less pollution and more efficient production methods that may eventually enhance a firm’s competitiveness. Those seminal papers define the so-called “Porter” hypothesis, which establishes arguments against the traditional trade-off between improving the environment and other economic targets. Hart [1] refers to these arguments from a natural resource–based view of the firm, claiming that competitive advantages should be based on key capabilities that facilitate environmentally sustainable economic activities and linking the development of such capabilities to positive economic returns. He argues that capabilities such as “pollution prevention”, “product management” and “sustainable development” provide competitive advantages by offering cost reductions, anticipating competitors and improving stakeholder relations.

This point of view was later adopted in several studies that analyze how the accumulation of positive resources and capabilities associated with environmental protection improves competitiveness (Russo and Fouts [3], Sharma and Vredenburg [5], Bansal [39], Walls et al. [12]). However, more than two decades after the Porter hypothesis, there is no consensus on the relationship between economic and environmental performance.

One of the key concepts that might help to clarify this debate is environmental proactivity, as it has been highlighted for facilitating a positive relationship between environmental protection and economic performance [34].

Based on the definitions proposed by authors such as Berry and Rondinelli [40], Sharma and Vredenburg [5], Aragón-Correa and Sharma [30] and Murillo et al. [41], in this paper we define environmental proactivity as s firms’ tendency to apply environmental protection measures in a voluntary way, anticipating environmental requirements coming from regulations and stakeholders and surpassing regulatory compliance and the measures generally adopted in the industry.

Russo and Fouts [3] have warned about the different financial consequences of a “compliance strategy”, whereby a firm adopts an “end-of-pipe” approach based on pollution abatement for basic compliance with environmental legislation. This is in contrast with a “go-beyond-compliance” strategy, whereby a firm adopts a preventive approach that emphasizes source reduction and process innovation. Following Russo and Fouts [3], firms that tend towards a compliance mode differ in their resource bases from those that tend toward prevention, and this difference conditions a firm’s ability to generate profits. Taking on the resource-based view, these authors explain that the physical assets and technology required for end-of-pipe compliance policies, such as filters or purification equipment at the end of the production process, are easily imitated and do not require the firm to develop expertise or skills. However, the replacement of equipment by new and more efficient models associated withgo-beyond-compliance strategies requires know-how and internal improvements that are not easily imitated. Regarding the human resources required for environmental strategies, Russo and Fouts [3] maintain that go-beyond-compliance is a more comprehensive and socially complex process than compliance, as it requires more employee involvement and greater cultural, integration, communication and coordination capabilities. Finally, Russo and Fouts [3] have also shown the advantages of going beyond compliance to accumulate intangible resources in the form of reputation and the ability to influence legislators and public policy.

Klassen and Why bark [23] and King and Lenox [25] have supported the above arguments with empirical evidence. They found strong evidence that, unlike reactive strategies based on “end-of-pipe” pollution control, prevention-based environmental protection strategies improve financial performance. González-Benito and González-Benito [26] and Aragón-Correa et al. [27] have also concluded that firms with more proactive environmental practices improve their financial performance to a greater extent.

More recently, Ghisetti and Rennings [29] empirically showed that environmental innovations aimed at increasing energy and resource efficiency lead to a potential “win-win” situation, in which reducing the environmental impact of production enhances economic performance. However, with innovations that merely reduce externalities (such as harmful materials, air pollution, etc.), it does not pay to be green.

These arguments, together with those established in the last decade by authors such as Guenster et al. [10], Fujii et al. [42], Sambasivan [28] and Trumpp and Guenther [34] have shown that the traditionalist “it costs to be green” view relates to end-of-pipe corrective technologies and reactive strategies adopted to respond to compulsory requirements, while the revisionist “it pays to be green” view relates to preventive technologies for cleaner production and proactive strategies that go far beyond compliance-based practices.

Consistent with the above arguments, this study refers to environmental proactivity as a key element in the possibility of generating associated economic returns. In order to analyze that relationship, we need to develop an objective measure of environmental proactivity. Most previous studies have been based on primary information (surveys or interviews) to measure environmental proactivity. Some of these studies group together different variables or items that represent environmental practices in latent factors or variables, evaluating firms according to factor scores (for instance, Aragón et al. [30]). However, most authors who measure proactivity from a qualitative perspective classify firms in a discrete number of categories or clusters according to type of environmental practice (for example, Aragón-Correa [43]; Sharma and Vredenburg [5]; Henriques and Sadorsky [44]; Gonzalez-Benito and González-Benito [26]; Murillo et. al. [41] and Sambasivan [28]).

This study proposes an environmental proactivity measure, calculated from objective secondary information, that enables a comparison of the environmental proactivity of firms of different sizes from a range of sectors. Once this variable, called the Environmental Proactivity Index (EPI), has been defined, this study attempts to analyze whether companies with a greater degree of environmental proactivity are more highly valued by the market.

We thus propose the following hypothesis:

**H1.**  *The greater a firm’s degree of environmental proactivity, defined as the tendency to go beyond compliance and institutionalized environmental practices in the industry, the better its financial performance.*

## 3. The Moderating Effect of External Situational Factors: A Contingent Theory Approach

Different authors have highlighted the possible influence of external situational factors in studies of the economic consequences of environmental proactivity. Aragón-Correa and Sharma [30], using contingency theory arguments proposed by Lawrence and Lorsch [45], showed the convenience of considering these moderating effects. According to contingency theory, a coherent alignment of internal organizational variables with exogenous context variables will result in superior organizational performance. As a result, it is crucial to match organizational resources with opportunities and threats in a general business environment [46]. Therefore, empirical literature has suggested a number of moderating factors in the study of the economic consequences of environmental proactivity such as: industrial growth [3]; uncertainty, complexity and munificence [30]; degree of strictness of environmental requirements [16,31]; the presence of green external economic incentives [47]; interdependence among certain external contingencies and organizational life cycle stages [48]; and the uncertainty perceived by managers in a firm’s business environment [49]. In the context of our research, there are two new and important aspects that could have a significant effect on the relationship between environmental proactivity and economic performance: the EU Emission Trading Scheme (EU ETS) and the financial crisis. 

### 3.1. The EU Emission Trading System (EU ETS)

The EU Emission Trading System (EU ETS), regulated through Directive 2003/87/EC, represents the most important mechanism in the reduction of greenhouse gas (GHG) emissions by the European Union in order to achieve 2020 EU targets. This environmental regulation mechanism affects the production facilities of the largest carbon-intensive industry sectors. The EU ETS sets a cap that represents the total limit of allowed GHG emissions, and divides this total amount into a number of emission permits that are distributed among companies. The possibility of buying and selling these permits in a market designed for this purpose gives flexibility to companies when choosing the optimal level of pollution reduction. One advantage of the EU ETS relative to other environmental policy mechanisms is cost effectiveness, as it allows each company to choose its individual optimal level of pollution reduction. Although the price of CO_2_ emission permits may be conditioned by macroeconomic factors outside the GHG market [50], the establishment of a price for CO_2_ is expected to generate continuous dynamic incentives for GHG mitigation technologies [51]. However, companies must consider that the implementation of effective GHG mitigation technologies can be very complex, and often demands long lead times and substantial capital expenditures [52,53]. Thus, while the reduction of emissions in substances such as SOx-NOx can be achieved through end-of-pipe technologies, CO_2_ abatement requires more radical changes to companies’ productive processes [54,55] and, in certain energy intensive industries, GHG mitigation might require substantial changes in an energy mix [56,57].Therefore, it can be said that the implementation of the EU Emission Trading System applies considerable pressure on firms to adapt their production processes in order to remain both competitive and sustainable, and affects the possibilities of finding a win-win situation as the results of a proactive environmental strategy.

This effect is considered through the following hypothesis:

**H2.**  *Regulatory pressure from the EU ETS moderates the improvement of financial performance derived from environmental proactivity.*

### 3.2. The Financial Crisis

The other exogenous variable that could be seen as having a significant effect on the relationship between environmental proactivity and economic performance is the global financial crisis, which began in 2008. Major economic shocks are often associated with high levels of uncertainty in demand conditions and among new business opportunities. In such economic conditions, organizations become less willing to invest in long-term activities where returns are risky. That is precisely the case for investments in Research and Development (R&D) and innovation projects. A large number of studies have shown that the financial crisis affected firms’ investment efficiencies [58], and forced many firms to reduce innovation investments [59,60,61,62]. Although the impact of an economic crisis on environmental innovation investment has been less studied, some papers have suggested that, in times of crisis, companies’ strategic behaviors become more conservative and defensive, which translates into a decrease in investments in sustainability [63,64,65]. Therefore, it can be presumed that the uncertainty posed by the financial crisis has conditioned the level of investment in environmental protection, and could have moderated the possibility of obtaining positive economic results derived from a proactive environmental strategy. 

We thus propose the following hypothesis:

**H3.**  *The financial crisis has moderated the improvement of financial performance derived from environmental proactivity.*

## 4. Research Framework 

The empirical analysis conducted in this study is based on information obtained from three sources: the public annual reports of the firms registered in the Spanish National Stock Market Commission (Comisión Nacionaldel Mercado de Valores (CNMV)), the Sistema de Análisis de Balances Ibéricos (SABI) database and the information about listed firms published by the Madrid Stock Exchange database.

In this study we propose an innovative design of the variable that measures environmental proactivity. The main objective of this design is to take into account one of the main aspects associated with the term proactivity, and in particular the level at which the environmental effort made by companies exceeds the average level in a sector. As a result, we propose a continuous and objective measure called the Environmental Proactivity Index (EPI), calculated from objective secondary information: firms’ environmental investments. 

Public information on environmental investment has been available in the annual reports of listed Spanish firms since 2002 (Commission Recommendation 2001/453/EC). Therefore, a sample of 42 industrial firms listed on the Madrid Stock Exchange Continuous Market was selected, and a panel database was generated with observations from the 2005–2012 period. The 42 companies in the sample belonged to different sectors (see Table 1) and represented around 75% of all listed Spanish industrial firms in the considered period.

The Environmental Proactivity Index (EPI_it_) corresponding to firm *i* during period *t* is calculated as follows:

(1) $${\mathrm{EPI}}_{\mathrm{it}}=\frac{{\mathrm{ETC}}_{\mathrm{it}}}{{\mathrm{Firm}\;\mathrm{Turnover}\;}_{\mathrm{it}}}-{\mathrm{EIE}}_{s}$$

where ETC_it_ is the Environmental Technology Capital that the company has accumulated in the last five years; the $\frac{{\mathrm{ETC}}_{\mathrm{it}}}{{\mathrm{Firm}\;\mathrm{Turnover}}_{\mathrm{it}}}$ ratio represents the environmental investment effort made by company *i* in period *t* (EIE_it_); and EIE_s_ is the mean environmental investment effort of companies in the sector in which company *I* operates.

Following Pakes and Schankerman [66] a firm’s ETC_it_ is calculated using a stock measure constructed from a formulation of depreciated sums of the environmental investments made in the last few periods, using Koyck lags:

(2) $${\mathrm{ETC}}_{\mathrm{it}}=\sum_{p=0}^{P}{\left(1-\delta \right)}^{P}{\mathrm{Environmental}\;\mathrm{Investment}\;}_{i,t-p}$$

where δ is the depreciation rate, and *P* is the number of years before the current year in which environmental investments affected the stock of Environmental Technology Capital. we considered a depreciation rate of 20% and a useful life of five years for environmental investments, as Hirschey and Weygandt [67] and Henderson and Cockburn [68] determined for R&D investments. 

Environmental investment _i,t–p_ refers to “elements added to the organization’s long-term assets, the primary purpose of which is to minimize environmental impact and protect the environment, including the reduction or elimination of future pollution arising from the organization’s operations”, according to the Spanish Institute of Accounting and Auditing (ICAC Resolution of 25 March 2002).

The firm turnover _it_ variable is used in the calculation of the Environmental Proactivity Index (EPI_it_) in order to consider the relationship between company size and innovation, as a consequence of economies of scale involved in R&D activities [69] and the lower risk involved in larger firms [70].

Finally, sectoral effects are considered by subtracting mean sectoral effects (*EIE_s_*) from each individual environmental investment effort (EIE_it_). This subtraction enables us to consider the extent to which each company’s individual effort exceeds the average effort in the sector.

Additionally, two dummy variables were created: “dummy crisis” and “dummy ETS”.

Dummy crisis records a value of 1 for the years of the crisis period (2009 to 2012), and a value of 0 for every year outside the crisis period (2005–2008).

Dummy ETS recordsa value of1 for firms that must participate in EU ETS, or a value of 0 otherwise.

The dependent variable is Tobin’s q, which measures the economic performance of a firm as follows:

(3) $${\mathrm{Tobin}}^{\prime }s\;q=\frac{\mathrm{Market}\;\mathrm{Value}\;\mathrm{of}\;\mathrm{the}\;\mathrm{Firm}\;(\mathrm{MV})}{\mathrm{Replacement}\;\mathrm{Cos}t\;\mathrm{of}\;\mathrm{Tan}\mathrm{gible}\;\mathrm{Assets}\;(\mathrm{RC})}$$

Tobin’s q refers to the expectations investors hold about a firm’s capacity to generate future economic benefits [71].

Table 2 presents descriptive statistics and correlations. 

By using panel data econometrics, we can distinguish between fixed- and random-effects estimation models, depending on how each specific effect is accounted for. The first assumes that there is a different constant term for each individual, and that individual effects are independent from one another. In the random-effects model, however, individual effects are not independent, but randomly distributed around a given value. The convenience of treating specific effects one way or the other depends on whether or not they are correlated with observable variables. 

Panel data econometrics provides advantages such as an increase in the number of observations, an increase in degrees of freedom or a reduction of co-linearity between exogenous variables [72].

## 5. Results

Both in the random-effects and fixed-effects estimations, two types of model were considered: the one-way model, which controls for firm-specific effects; and the two-way model, which controls for both firm-specific and time-specific effects.

Breusch–Pagan and Hausman test results showed the random-effects estimation was the most efficient. Therefore, we selected the random-effects estimated models. Moreover, we performed a Mundlak test [73] to relax the assumption that explanatory variables are non-correlated with unobserved variables. The Mundlak test results confirmed that random-effects estimators were the most efficient in models 1 to 4. However, the Mundlak test was significant in model 5, so in this case the fixed-effects model represented the best specification.

### 5.1. Effect of Environmental Proactivity on Economic Performance

According to the results shown in Table 3, Hypothesis 1 was not rejected, suggesting that the greater a firm’s degree of environmental proactivity, the greater its financial performance. Indeed, one-way model 1 showed that the Environmental Proactivity Index (EPI) had a positive and significant effect (*p*-value < 0.05) on the dependent variable (Tobin’s q).

Two-way model 1 showed that the Environmental Proactivity Index (EPI) was positive and significant at a1% level, and that the positive effect of environmental proactivity on economic performance was maintained even when heterogeneity was controlled across time periods. There is evidence that time-specific effects were are jointly significant, suggesting that they should have been they should be included in the model.

Subsequently, the two contingent variables (dummy ETS and dummy crisis) were included in the model as control variables.

Model 2 showed that the coefficient of the dummy ETS variable was not statistically significant, and that belonging to the EU ETS made no significant difference to firms’ economic results. This lack of significance was confirmed when time-specific effects were included in the model.

Model 3 included a dummy variable (dummy crisis) that considered the possible effect of the financial crisis (2009–2012) on firms’ economic performances. 

The results of the model indicated that the coefficient of the dummy crisis was negative and significant at a1% level, showing that the economic performances of the firms were worse during the crisis than they were before. 

In sum, the results provide additional empirical evidence that firms with greater environmental proactivity have better economic results, and that this positive effect is maintained even when controlling the possible influence by the financial crisis and regulatory pressure from the EU ETS.

### 5.2. Analysis of the Moderating Effect of the Contingent Variables

Hypothesis 2 suggests that forming part of the EU ETS moderates the improvement of economic performance derived from environmental proactivity. To test this hypothesis, an interaction term was included that was the result of multiplying the Environmental Proactivity Index by the dummy ETS variable (EPI x dummy ETS) (As shown in model 4 (Table 4), the results suggest that Hypothesis 2 was not rejected. The interaction term (EPI x dummy ETS) was negative and significant at the 1% level. Furthermore, the R^2^ of the model increased from 0.014 without the interaction term (model 2), to 0.060 with the interaction term (model 4). This specifically showed that investment in environmental proactivity had a lesser effect on economic performance in firms subject to regulatory pressure from the EU ETS. This result was confirmed even when heterogeneity was controlled across time periods (two-way model 4). 

Hypothesis 3 suggests that the financial crisis has moderated the improvement of economic performance derived from environmental proactivity. To test this hypothesis, an interaction term was included (EPI x dummy crisis). As model 5 shows, the data do not support this hypothesis. The coefficient of the interaction term (EPI x dummy crisis) was not significant, showing that the crisis did not moderate the impact of environmental proactivity on economic performance. 

## 6. Conclusions

This study adds empirical evidence to what is known about the relationship between environmental investment efforts and financial results, and considers some of the conditions and circumstances in which it pays to be green. The research concludes that a proactive attitude in environmental strategy (the tendency to voluntarily apply environmental protection measures to a greater extent than required by law or is common in a firm’s sector) contributes to obtaining associated economic benefits. The study obtains empirical evidence that firms that invest more than the sector average towards environmental protection are more highly valued by the market. These results confirm the idea that it pays to go beyond compliance in environmental management, established by authors such as Russo and Fouts [3]; Klassen and Why bark [23]; King and Lenox [25]; and Ghisetti and Rennings [29], among others. 

This research also concludes that the economic results generated by environmental investment can be conditioned by external factors and situations. First, this study reaches conclusions regarding the impact of the global financial crisis on the possibility of obtaining a win-win situation with a proactive environmental strategy. In this regard, it is concluded that a win-win situation is guaranteed even during a global financial crisis. Indeed, while firms’ economic performances were worsened during the crisis, this did not apply to the benefits of environmental proactivity. These results are consistent with those found by authors such as Fujii et al. [42], whose study of Japanese manufacturing firms also concludes that, although the financial crisis had a negative impact on technical efficiency, it did not affect environmental efficiency. 

On the other hand, although less environmental investment can be expected in a recession, it is expected of all sector firms, so the effect on degree of proactivity (which measures the tendency to exceed mean investment effort in the sector) is smaller. 

Finally, this research concludes that while firms affected by regulatory pressure from the European Union Emissions Trading System (EU ETS) do not have a worse economic performance than other firms, they find their environmental proactivity less useful. To explain this, we have to consider the nature of the investments required to reduce CO_2_ emissions. Unlike other pollutants that can be mitigated by end-of-pipe technologies, CO_2_ abatement depends upon a much more radical change in firm processes. This radical transformation, however, is not widespread among ETS firms, or at least not enough to make full use of environmental investments. Some studies show a rather limited innovation impact of the EU ETS, particularly on research and development and long-term portfolio decisions [74,75,76,77,78]. This could be due to a lack of stringency in the first (2005–2007) and second (2008–2012) trading phases, when too many free permits were granted and no consideration was given to the fall in activity caused by the economic crisis that began in 2008. As a result, allowance prices have been volatile and sometimes very low, therefore resulting in a weak incentive to implement energy efficiency and non-emitting technologies. Thus, a possible reason for the smaller impact of environmental proactivity in ETS firms could be the inefficient design of the EU ETS mechanism, which fostered investments that, as mentioned by Gilli [55], were more incremental than radical. Those incremental investments were insufficient, and did not generate the conditions of anticipation and flexibility required to favor the full efficacy of investments in environmental proactivity. 

On the other hand, the adaptation of production processes to reduce CO_2_ emissions can be very costly. This cost is comparatively higher in ETS firms, which could explain why environmental investment in such firms has lower economic results. 

Our conclusions are of academic interest as they teach us more about the economic consequences of environmental effort, and identify some of the contingencies and situations in which it pays to be green. This study is also useful for business managers as it demonstrates that, when designing environmental strategy, it pays both to go beyond compliance and regular sector practices and to appropriately integrate them in overall business strategy. From the perspective of policy-makers, their fundamental challenge is to create enough incentives for the companies participating in the EU Emission Trading System to influence their investment decision-making. Companies must be prepared for the likelihood of stricter EU ETS conditions in the short and medium terms. On 27 February 2018 the European Council approved the reform of the EU Emissions Trading System (ETS) for the period after 2020. The reform ultimately aims to push CO_2_ prices up. In a context of higher prices, only firms that develop an anticipation strategy, through radical changes in their production process aimed at reducing CO_2_ emissions, can be expected to improve their competitive positions relative to firms with more conservative environmental strategies.

## Figures and Tables

**Table 1:** Distribution by sector.

Industrial Sector	No. Firms	No. Obs.
Oil and energy	7	56
Minerals, metals and processing	5	40
Mechanical, electrical and electronic equipment	6	48
Construction	3	24
Cement	2	16
Chemical and paper	6	48
Pharmaceutical products and biotechnology	5	40
Food and beverages	6	48
Textile and footwear	2	16
**TOTAL**	**42**	**336**

**Source:** Based on the Madrid Stock Exchange’s classification.

**Table 2:** SDs and correlations for quantitative variables.

Variables	Description	Mean	SD	1	2	3	4
**Tobin’s q**	Market value of firm/replacement cost of tangible assets	1.853	1.055	1.000			
**Environmental Proactivity** **Index (EPI)**	(environmental technology capital (ETC) accumulated by the firm in the last 5 years/size)—mean environmental investment effort (EIE) of the sector	−0.004	0.028	0.109	1.000		
**Dummy crisis**	Dummy = 1 for each crisis years (2009–2012), or 0 for each year before the crisis (2005–2008)	0.500	0.500	−0.267	0.064	1.000	
**Dummy ETS**	Dummy = 1 for EU ETS firms or 0 otherwise	0.452	0.498	−0.185	−0.106	0.000	1.000

**Table 3:** Panel estimation. Hypothesis 1.

Independent Variables	DEPENDENT VARIABLE: Tobin’s q									
	Model 1 (One-Way)		Model 1 (Two-Way)		Model 2 (One-Way)		Model 2 (Two-Way)		Model 3 ^1^ (One-Way)	
	Fixed	Random	Fixed	Random	Fixed	Random	Fixed	Random	Fixed	Random
Constant	1.873 (0.000)	1.872 (0.000)	1.523 (0.000)	1.522 (0.000)	1.873 (0.000)	2.037 (0.000)	1.523 (0.000)	1.683 (0.000)	2.173 (0.000)	2.171 (0.000)
Environmental Proactivity Index (EPI)	4.454 ** (0.041)	4.371 ** (0.031)	6.110 *** (0.001)	5.897 *** (0.001)	4.454 ** (0.041)	4.227 ** (0.037)	6.110 *** (0.001)	5.789 *** (0.001)	6.119 *** (0.002)	5.876 *** (0.002)
Dummy ETS firms					-	−0.365 (0.148)	-	−0.356 (0.159)		
Dummy crisis									−0.586 *** (0.000)	−0.585 *** (0.000)
Time effects D_2005_			0.651 ***	0.651 ***			0.651 ***	0.651 ***		
D_2006_			0.944 ***	0.943 ***			0.944 ***	0.943 ***		
D_2007_			0.903 ***	0.901 ***			0.903 ***	0.901 ***		
D_2008_			0.100	0.099			0.100	0.098		
D_2009_			0.107	0.107			0.107	0.107		
D_2010_			0.147	0.146			0.147	0.146		
D_2011_			0.000	0.000			0.000	−0.000		
No. observations	336	336	336	336	336	336	336	336	336	336
*R-squared*	0.014	0.014	0.333	0.333	0.014	0.014	0.333	0.333	0.203	0.203
Wald χ ^2^		4.65 (0.03)		143.73 (0.00)		6.75 (0.03)		145.64 (0.00)		74.86 (0.00)
F statistic	10.51 (0.00)		15.20 (0.00)		10.51 (0.00)		15.20 (0.00)		12.98 (0.00)	
Breusch–Pagan (χ^2^) (OLS vs. random)	339.67 (0.00)		481.25 (0.00)		320.94 (0.00)		460.63 (0.00)		415.40 (0.00)	
Hausman test (χ^2^) (random vs. fixed)	0.01 (0.91)		0.15 (0.70)		0.09 (0.76)		0.33 (0.56)		0.14 (0.93)	
Mundlak test (χ^2^)	0.02 (0.90)		0.23 (0.63)		0.18 (0.67)		0.94 (0.33)		0.23 (0.63)	

*p*-values are in parentheses. * *p* < 0.10, ** *p* < 0.05, *** *p* < 0.01. **^1^** There is no differentiation between the one-way and two-way models for model 3. The model of the dummy crisis for the 2009–2012 period controls for time-specific effects, making estimation of individual constant terms for each time period (D2005–D2011) redundant.

**Table 4:** Panel estimation. Hypotheses 2 and 3.

Independent Variables	DEPENDENT VARIABLE: Tobin’s q					
	Model 4 (One-Way)		Model 4 (Two-Way)		Model 5 (One-Way)	
	Fixed	Random	Fixed	Random	Fixed	Random
Constant	1.870 (0.000)	2.060 (0.000)	1.535 (0.000)	1.708 (0.000)	2.178 (0.000)	2.178 (0.000)
Environmental Proactivity Index (EPI)	19.168*** (0.000)	18.499 *** (0.000)	12.537 *** (0.001)	12.415 *** (0.001)	6.981 *** (0.001)	6.979 *** (0.000)
Dummy ETS firms	-	−0.419 (0.101)	-	−0.382 (0.134)		
Dummy crisis					−0.598 ***(0.000)	−0.600 ***(0.000)
*Interactions*						
EPI x dummy ETS firms	−19.080 *** (0.000)	−18.230 *** (0.000)	−8.454 * (0.056)	−8.599 ** (0.042)		
EPI x dummy crisis					−3.659 (0.215)	−4.303 (0.138)
*Time effects*						
D_2005_			0.630 ***	0.630 ***		
D_2006_			0.909 ***	0.907 ***		
D_2007_			0.867 ***	0.864 ***		
D_2008_			0.084	0.082		
D_2009_			0.104	0.103		
D_2010_			0.143	0.143		
D_2011_			0.005	0.005		
No. observations	336	336	336	336	336	336
*R-squared*	0.060	0.060	0.342	0.341	0.207	0.207
Wald χ ^2^		21.24 (0.02)		21.24 (0.02)		77.04 (0.00)
F statistic	11.07 (0.00)		15.31 (0.00)		12.69 (0.00)	
Breusch–Pagan (χ^2^) (OLS vs. random)	334.79 (0.00)		463.35 (0.00)		401.36 (0.00)	
Hausman test (χ^2^) (random vs. fixed)	0.33 (0.84)		0.17 (0.91)		1.92 (0.59)	
Mundlak test (χ^2^)	1.59 (0.45)		0.72 (0.69)		7.54 (0.02)	

*p*-values are in parentheses. * *p* < 0.10 ** *p* < 0.05 *** *p* < 0.01.
